# Modified lingguizhugan decoction incorporated with dietary restriction and exercise ameliorates hyperglycemia, hyperlipidemia and hypertension in a rat model of the metabolic syndrome

**DOI:** 10.1186/s12906-017-1557-y

**Published:** 2017-02-28

**Authors:** Limei Yao, Jingjing Wei, Si Shi, Kunbin Guo, Xiangyu Wang, Qi Wang, Dingsheng Chen, Weirong Li

**Affiliations:** 1 School of Traditional Chinese Medicine Healthcare, Guangdong Food and Drug Vocational College, 321 Longdong North Road, Tianhe District, Guangzhou, 510520 China; 2 Institute of Clinical Pharmacology, Guangzhou University of Chinese Medicine, 12 Jichang Road, Baiyun District, Guangzhou, 510405 China

**Keywords:** Metabolic syndrome, Modified lingguizhugan decoction, Dietary restriction, Exercise, High-fat diet

## Abstract

**Background:**

Modified Lingguizhugan Decoction (MLD) came from famous Chinese medicine Linggui Zhugan Decoction. The MLD is used for the treatment of metabolic syndrome in the clinical setting. Our study focuses on the comprehensive treatment of MLD incorporated with dietary restriction and exercise in a rat model of the metabolic syndrome (MS).

**Methods:**

Rats were divided into five groups: control group (Cont), high-fat diet group (HFD), high-fat diet incorporated with dietary restriction group (HFD-DR), exercise incorporated with dietary restriction group (HFD-DR-Ex) and MLD incorporated with dietary restriction and exercise group (HFD-DR-Ex-MLD). Treatments were conducted for 1 week after feeding high-fat diet for 12 weeks. The effects of treatments on high fat diet-induced obesity, hyperglycemia, hyperlipidemia, hypertension, hepatic injury and insulin resistance in rats of MS were examined. In addition, the tumor necrosis factor-α (TNF-α), leptin and protein kinase B (PKB) in rats serum and liver were also examined by enzyme-linked immunosorbent assay (ELISA).

**Results:**

After a week’s intervention by dietary restriction, dietary restriction incorporated with exercise or MLD, compared with HFD rats, the relative weight of liver and fat, levels of triglyceride, total cholesterol, low-density lipoprotein, free fatty acid, aspartate aminotransferase, glutamic-pyruvic transaminase and alkaline phosphatase, insulin, were significantly decreased (*p* < 0.05 or 0.01). This treatment also inhibited abnormal increases of TNF-α, leptin and PKB in serum and liver.

**Conclusion:**

MLD incorporated with dietary restriction and exercise treatment exhibit effects in alleviating high-fat diet-induced obesity, hyperglycemia, hyperlipidemia, hypertension, hepatic injury and insulin resistance, which are possibly due to the down-regulation of TNF-α, leptin and PKB.

## Background

Metabolic syndrome (MS) is a chronic systemic disorder disease characterized by abdominal obesity, abnormal glucose metabolism, blood lipid disorders, high blood pressure and cardiovascular disease [1, 2]. MS is a leading cause of mortality and morbidity in industrialized countries [3]. In the United States, national surveys have observed that the prevalence of MS was 23.7% in the general population [4]. In China, a survey of 35–74 year-old adults showed that the prevalence of MS was 10.0 and 23.3% for males and females, respectively [5].

MS is a disease composed of different risk factors such as obesity, type 2 diabetes or dyslipidemia. The prevalence of this syndrome is increasing worldwide in parallel with the rise in obesity and indicates that greater attention should be paid to it [6]. Obesity is now a worldwide pandemic and is expected to affect 10% of the global population by 2030 if the current trend is maintained [7]. Obesity results from an imbalance between caloric intake and energy expenditure, leading to an excess of energy, which is stored as fat mainly in white adipose tissue [8]. It is well recognized that inappropriate diet and inactivity are important determinants of MS and other chronic disorders. Dietary restriction and exercise have drawn attention in recent years due to its effect on weight loss to reduce the risk factors of cardiovascular disease in obese patients [9]. Clinical and experimental evidence showed that diet restriction and exercise can be a very effective strategy to prevent the development of a metabolic syndrome [10, 11].

Traditional Chinese medicine (TCM) has its unique advantages in preventing these diseases. Linggui Zhugan Decoction (LZD) was a famous Chinese medicine formulae which was documented in *Jin Gui Yao Lue*, a classical work of Zhongjing Zhang written in the *Han* dynasty. Zhang JJ et al. observed alternate-day fasting therapy combined with LZD could reduce body weight, total cholesterol (TC), triglyceride (TG) concentrations and elevate glutathione in hyperlipidemic rats [12]. LZD combined calorie restriction could reduce the body weight, fasting plasma glucose, and insulin resistance index of insulin resistance rats [13]. The Modified Lingguizhugan Decoction (MLD) came from LZD and showed better efficacy. MLD can lower body weight, blood pressure (BP), blood glucose, the level of TC, TG and low-density lipoprotein (LDL), elevate high-density lipoprotein (HDL), regulate adiponectin, leptin, insulin and insulin resistance index to a normal level in MS model rats, which suggested that MLD has effects on regulation fat and saccharide metabolism [14–16]. The MLD incorporated with short-term diet restriction and exercise is a new kind of natural therapy incorporating Chinese medicine with fasting therapy systematically [17]. The treatment requires the participants to have less solid food, but drink millet soup. The MLD with the effects of warming and resolving the mucus, strengthening the spleen and removing the turbidity is simultaneously administered to restore normal function of the spleen in transportation and transformation [18, 19]. Therefore, this study investigated effects of dietary restriction, dietary restriction incorporated with exercise, dietary restriction incorporated with exercise and MLD on high-fat diet-induced MS in rats.

## Methods

### Preparation of MLD

**Fig. 1:**
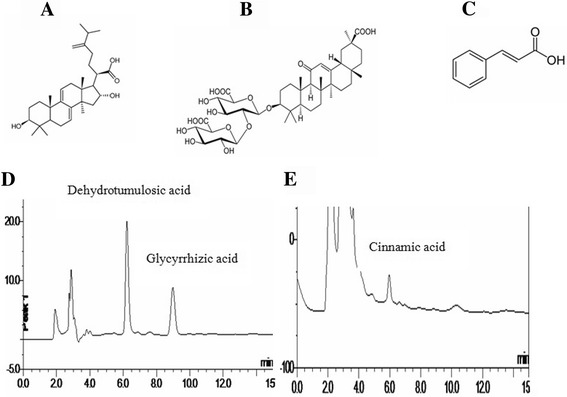
Chemical structures and HPLC analysis of major components in MLD. **a** dehydrotumulosic acid (C_31_H_48_O_4_, molecular weight: 484.71); **b** glycyrrhizic acid (C_42_H_62_O_16_, molecular weight: 822.93); **c** cinnamic acid (C_9_H_8_O_2_, molecular weight: 148.16); **d** HPLC analysis of dehydrotumulosic acid and glycyrrhizic acid in MLD; **e** HPLC analysis of cinnamic acid in MLD

MLD was composed of *Poria cocos* (Schw.) Wolf, *Cinnamomum cassia* Presl, *Atractylodes lancea* (Thunb.) DC., *Glycyrrhiza uralensis* Fisch., *Codonopsis pilosula* (Franch.) Nannf. and *Rheum palmatum* L at the ratio of 12:9:6:6:9:9. Crude drugs are offered by Sanjiu Medical and Pharmaceutical Incorporation (Shenzhen, China). All of these herbal materials were the same batch during the whole experimental process and were identified by School of Chinese Materia Medica, Guangzhou University of Chinese Medicine. A voucher specimen of each species was also deposited at Guangzhou University of Chinese Medicine. To prepare the MLD, the mixture of the six herbs were pulverized to coarse powder, refluxed in water two times each for 1 h. The extracts were combined and filtrated. The filtration were concentrated to 0.617 g/mL (crude drug content) and stored at 4 °C until use. To ensure the quality and stability of the MLD, we use high performance liquid chromatography (HPLC) to assay important efficacy components in MLD (dehydrotumulosic acid, cinnamic acid and glycyrrhizic acid). The concentrations of the three components were: dehydrotumulosic acid (75.87 μg/ml), cinnamic acid (78.36 μg/ml) and glycyrrhizic acid (247.21 μg/ml). The chemical structures and chromatograms are shown in Fig. 1.

### Animals

The study protocol was approved by the Institutional Animal Care and Use Committee of Guangzhou University of Chinese Medicine and in accordance with the principles outlined in the NIH Guide for the Care and Use of Laboratory Animals. Fifty adult Sprague-Dawley male rats (SPF grade) weighing 200 ± 20 g, 6- to 7-week-old, were kept in a temperature and humidity controlled animal facility and maintained on a 12 h light/dark cycle with access to food and water ad libitum. Experiments were conducted during the light phase. All rats had free access to diets and water for 1 week prior to the experiments. The diet was a semi-purified modified D12451 formulation with energy sources containing 36% energy and 1% cholesterol. The diet composition was similar to a normal human diet.

### Animal treatment

After 1 week of acclimatization, 50 rats were randomly divided into five groups with ten animals in each group. (1) Control group (Cont) that accepted normal chow (energy content: protein 23%, carbohydrate 67%, and fat 10%); (2) high-fat diet group (HFD) that fed with high-fat diet (energy content: protein 12%, carbohydrate 18%, and fat 70% including 8% from soya oil and 62% saturated fat from lard) for 12 weeks to induce metabolic syndrome model. This high-fat diet enriched in saturated fat and incorporated with moderate-to-low carbohydrate content has been previously used as an obesogenic (causing high adiposity) and a diabetogenic (inducing insulin resistance) food in rats [20]; (3) high-fat diet incorporated with dietary restriction group (HFD-DR) that fed with 30% of high-fat diet; (4) exercise incorporated with dietary restriction group (HFD-DR-Ex) adhered to swim for 45 min everyday on the basis of HFD-DR; (5) MLD incorporated with dietary restriction and exercise group (HFD-DR-Ex-MLD) that gave MLD (correspond to 6.17 g crude drug/kg body weight) by oral administration everyday on the basis of HFD-DR-Ex. Treatments were conducted for 1 week after feeding high-fat diet for 12 weeks.

After a week of treatments, all rats were anesthetized with an intraperitoneal injection of chloralic hydras (3.5 mg/kg). After sacrifice of the rats, blood samples were collected from the abdominal aorta into tubes; serum was separated by centrifugation at 3500 rpms for 10 min and was kept frozen at −80 °C until chemical assays. The epididymal and retroperitoneal fat pads were excised and weighed. The liver was also excised and weighed, then took a leaf of liver fixed with neutral formaldehyde for histological study, the rest was kept at −80 °C for ELISA.

### Exercise training

The exercise training protocol was adapted from a previously published procedure [21]. Swimming was done in a cylindrical tank, 180 cm in diameter and 50 cm in height, temperature (29 ± 2) °C, according to the protocol. The animals were placed in the tank daily at the same hour (11:00–11:45 A.M.), and the training was monitored by the same person. The rats swam for a week (45 min/day), after each exercise session, the animals were dried and kept in a warm environment. Other group rats were restricted to cage activity.

### Blood pressure

Systolic blood pressure was measured using a tail-cuff system (Shanghai Alcott Biotech Co., Ltd., China) before and at the end of the experimental period. All of the animals were placed in a restrainer for 15 min, a cuff was attached to their tail, and the blood pressure was recorded. For both measures, the rats underwent an adaptation period of 7 days to familiarize them with the procedure, which began right after the adaptation period. Valid blood pressure values were obtained as described at the 8th day of measurements.

### Body weight and relative weights of adipose tissue and liver

After sacrifice rats, the epididymal and retroperitoneal fat pads were excised and weighed. The liver was also cleaned and the wet weights were measured. Weights were calculated as percentage of wet weight to body weight.

### Biochemical determinations

Serum glucose was assayed by the fast blood glucose meter. Serum TG, TC, HDL, LDL, free fatty acid (FFA), aspartate aminotransferase (AST), glutamic-pyruvic transaminase (ALT) and alkaline phosphatase (ALP) were assayed using kits purchased from Nanjing Jiancheng Bioengineering Institute (Nanjing, China).

### Enzyme-linked immunosorbent assay (ELISA)

Serum was prepared from peripheral blood. Liver tissues were removed, homogenated with ice-cold PBS, centrifuged at 12,000 rpms for 20 min and separated the supernatant. The levels of insulin, leptin, protein kinase B (PKB) and tumor necrosis factor-α (TNF-α) in serum and liver tissue were measured by ELISA according to the manufacture instructions and calculated according to the standard curve.

### Histological study

An equal liver sample from each rat were obtained and fixed in 10% neutral formalin, dehydrated in ascending grades of alcohol and cleared in benzol. Samples from each group were embedded in paraffin with a melting point between 55 and 56 °C for 4 h and then paraffin blocks were prepared. Paraffin sections were made at 5 μm and stained with (1) hematoxylin and eosin (HE): for normal histology and histopathology; (2) periodic acid Schiff (PAS): to detect polysaccharides such as glycogen and mucosubstances such as glycoproteins, glycolipids and mucins in liver tissues. The Schiff reagent gives a purple-magenta color, followed by light microscopy for demons demonstrating any histological changes.

### Statistical analysis

All values are expressed as mean ± standard error of the mean (S.E.M.). Differences among groups were determined by one-way ANOVA with Tukey’s post hoc test. Statistical analyses were performed using SPSS version 11.0 (SPSS Inc., Chicago, USA). Statistical significance was confirmed when *P* values were less than 0.05 or 0.01.

## Results

### Body weight and relative weights of adipose tissue and liver

**Fig. 2:**
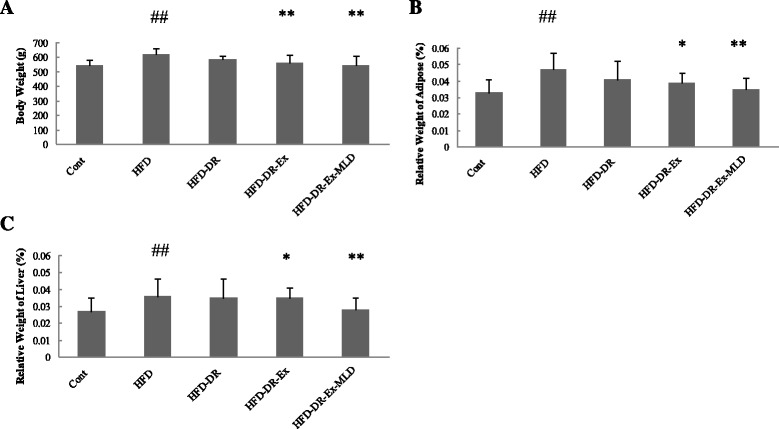
Changes in each group (*n* = 10) on body weight (**a**) and relative weight of adipose (**b**) and liver (**c**). Compared with Control group rats, ^##^ *P* < 0.01; compared with HFD rats, ^*^ *P* < 0.05, ^**^ *P* < 0.01, respectively

Figure 2 shows the body weight and relative weights of adipose tissue and liver after the intervention period. Compared with Cont rats, body weight (Fig. 2a) and relative weights of adipose tissue (Fig. 2b) and liver (Fig. 2c) of HFD rats was significantly increased (*P* < 0.01); compared with HFD rats, the body weight (Fig. 2a) and relative weight of adipose tissue (Fig. 2b) and liver (Fig. 2c) of HFD-DR-Ex and HFD-DR-Ex-MLD rats were significantly lower than the HFD rats (*P* < 0.05 or 0.01).

### Levels of TC, TG, LDL, HDL and FFA in serum

**Fig. 3:**
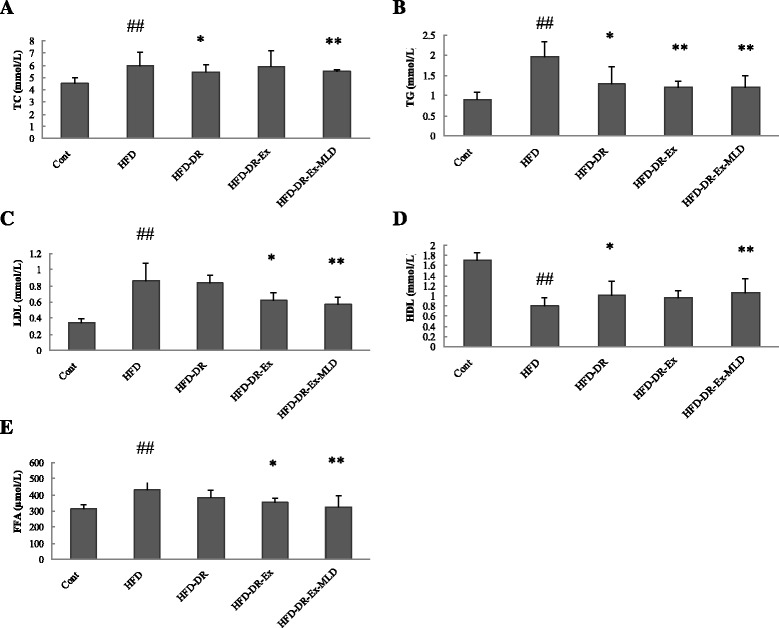
Changes in each group (*n* = 10) on TC (**a**), TG (**b**), LDL (**c**), HDL (**d**) and FFA (**e**). Compared with Control group rats, ^##^ *P* < 0.01; compared with HFD rats, ^*^ *P* < 0.05, ^**^ *P* < 0.01, respectively

The serum levels of TC, TG, LDL, HDL and FFA are demonstrated in Fig. 3. Compared with Cont rats, HFD rats resulted in significant increase in TC, TG, LDL and FFA and decrease in HDL (*P* < 0.01). Compared with HFD rats, levels of TC were significantly lower in HFD-DR-Ex-MLD rats (*P* < 0.01, Fig. 3a), levels of TG were significantly lower in HFD-DR, HFD-DR-Ex and HFD-DR-Ex-MLD rats (*P* < 0.05 or 0.01, Fig. 3b), levels of LDL were significantly lower in HFD-DR-Ex and HFD-DR-Ex-MLD rats (*P* < 0.05 or 0.01, Fig. 3c), levels of HDL were higher in HFD-DR and HFD-DR-Ex-MLD rats (*P* < 0.05 or 0.01, Fig. 3d), levels of FFA were significantly lower in HFD-DR-Ex and HFD-DR-Ex-MLD rats (*P* < 0.05 or 0.01, Fig. 3e).

### Levels of AST, ALT and ALP in serum

**Fig. 4:**
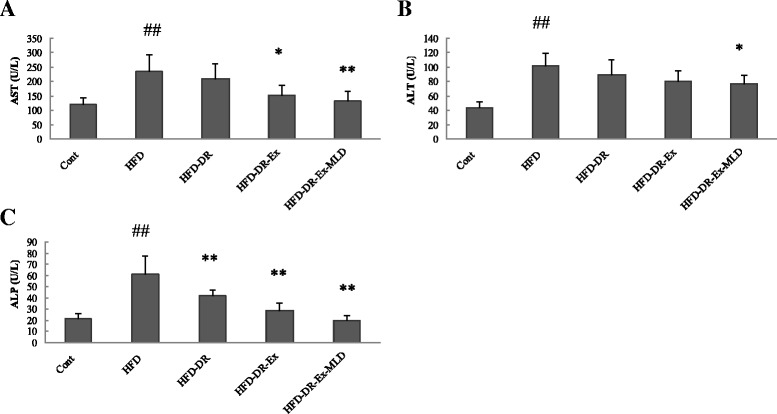
Changes in each group (*n* = 10) on AST (**a**), ALT (**b**) and ALP (**c**). Compared with Control group rats, ^##^ *P* < 0.01; compared with HFD rats, ^*^ *P* < 0.05, ^**^ *P* < 0.01, respectively

The serum levels of AST, ALT and ALP are demonstrated in Fig. 4. Compared with Cont rats, HFD rats resulted in significant increase in ALT, AST and ALP (*P* < 0.01). Compared with HFD rats, levels of AST (Fig. 4a) were significantly lower in HFD-DR-Ex and HFD-DR-Ex-MLD rats (*P* < 0.05 or 0.01), level of ALT (Fig. 4b) was significantly lower in HFD-DR-Ex-MLD rats (*P* < 0.05), levels of ALP (Fig. 4c) were significantly lower in HFD-DR, HFD-DR-Ex and HFD-DR-Ex-MLD rats (*P* < 0.01).

### Photomicrograph of rat’s liver

**Fig. 5:**
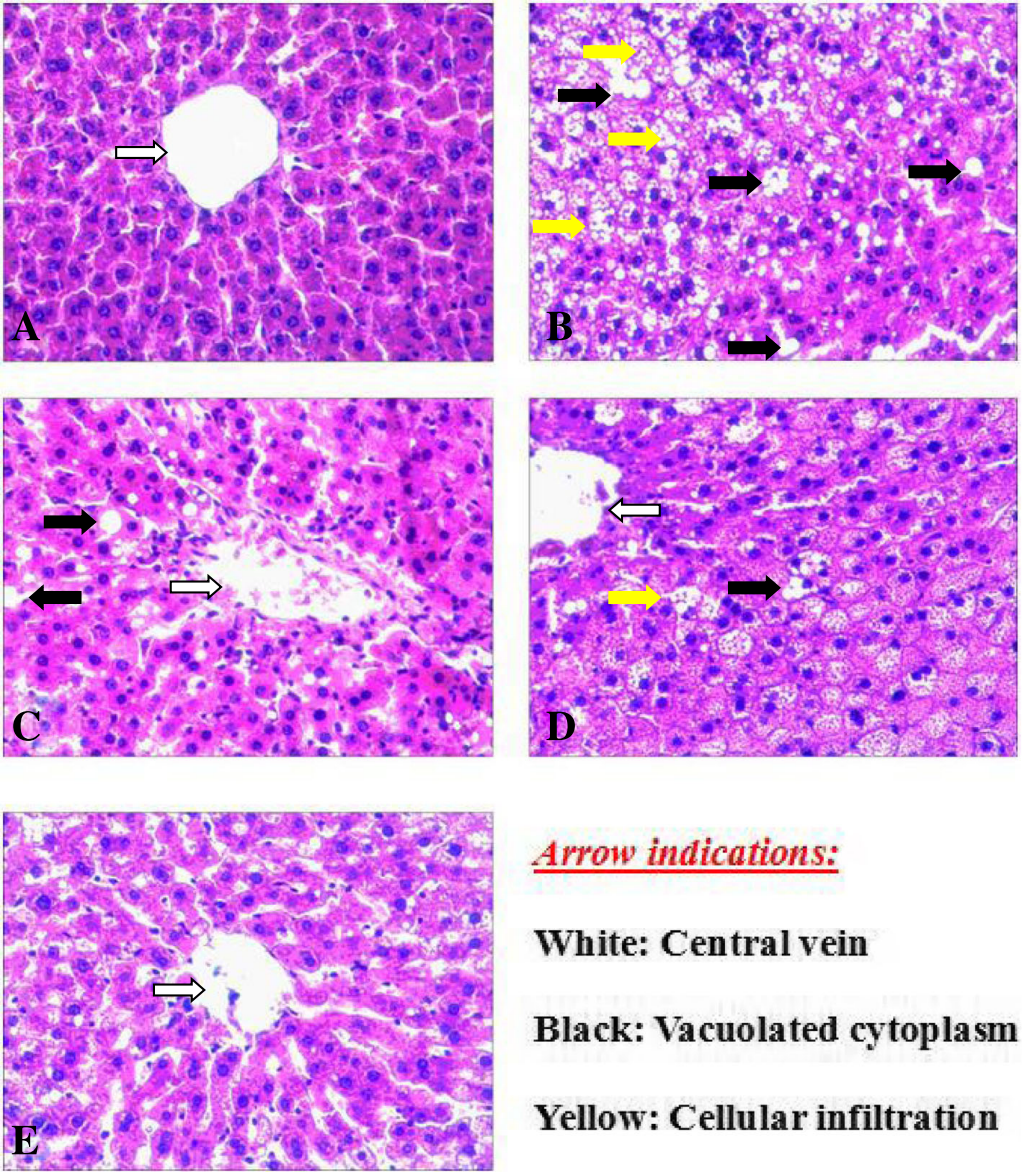
Photomicrograph of rats liver in Cont rats (**a**), HFD rats (**b**), HFD-DR rats (**c**), HFD-DR-Ex rats (**d**) and HFD-DR-Ex-MLD rats (**e**). (HE × 200)

As HE stain of rat’s liver revealed, Cont rats (Fig. 5a) histological architecture of liver showed normal lobular structure with central vein, hepatocytes and hepatic plates. Liver of HFD rats (Fig. 5b) showed serious periportal fibrosis, vacuolated cytoplasm and cellular infiltration. Liver of HFD-DR rats (Fig. 5c) showed somewhat normal liver cells and nuclei, compared to HFD group, vacuolated cytoplasm decreased significantly. Liver of HFD-DR-Ex rats (Fig. 5d) showed some cellular infiltrations. Liver of HFD-DR-Ex-MLD rats (Fig. 5e) showed small mean cell size, which was more or less similar to the levels of the Cont rats. There was an obvious difference in cell size distribution in each group.

**Fig. 6:**
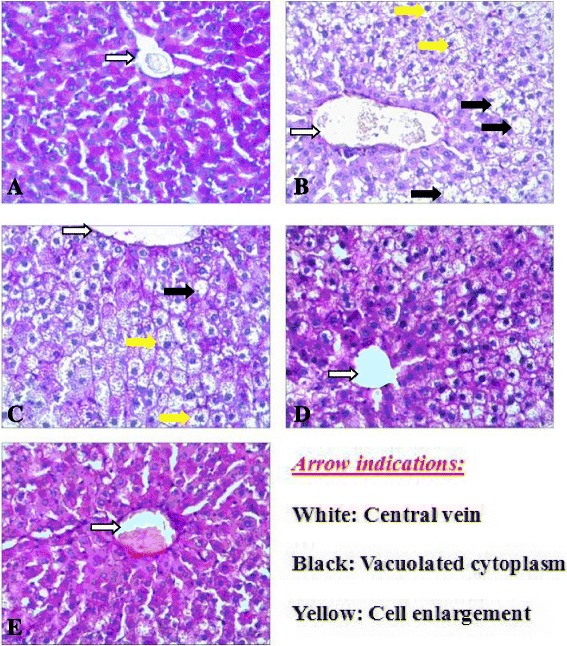
PAS stain of rats liver in Cont rats (**a**), HFD rats (**b**), HFD-DR rats (**c**), HFD-DR-Ex rats (**d**) and HFD-DR-Ex-MLD rats (**e**). (PAS × 200)

As PAS stain of rat’s liver revealed, Cont rats (Fig. 6a) shows normal architecture and glycogen distribution in the liver cell. Liver of HFD rats (Fig. 6b) showed changes in all hepatocytes. These cells became large with vacuolated cytoplasm and little red stained granules. Liver of HFD-DR rats (Fig. 6c) showed less cell enlargement and more red stained than HFD rats. Liver of HFD-DR-Ex rats (Fig. 6d) showed less hepatocytes and small cells with red stained granules than HFD-DR rats. Liver of HFD-DR-Ex-MLD rats (Fig. 6e) showed almost a normal liver architecture.

### BP, blood glucose and insulin

**Fig. 7:**
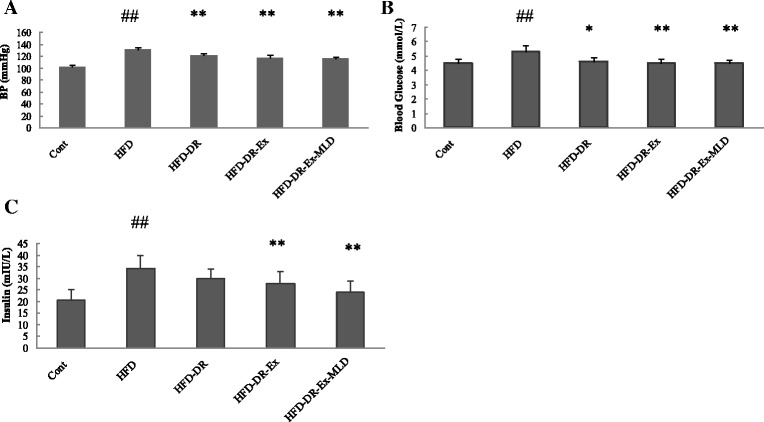
Changes of BP (**a**), blood glucose (**b**) and insulin (**c**) in each group (*n* = 10). Compared with Control group rats, ^##^ *P* < 0.01; compared with HFD rats, ^*^ *P* < 0.05, ^**^ *P* < 0.01, respectively

Before experimental period there was no difference on BP in each group (data not show). After the intervention period, compared with the Cont rats, The HFD rats showed a significant increase in BP (*P* < 0.01). Compared with the HFD rats, HFD-DR, HFD-DR-Ex and HFD-DR-Ex-MLD induced a significant decrease in BP (*P* < 0.01, Fig. 7a). Before and after the intervention, the blood glucose was measured by the fast blood glucose meter. Compared with the Cont rats, the blood glucose levels of HFD rats were significantly higher (*P* < 0.01, Fig. 7b); compared with the HFD rats, the blood glucose levels of HFD-DR, HFD-DR-Ex and HFD-DR-Ex-MLD were significantly lower (*P* < 0.05 or 0.01). The level of insulin in serum is demonstrated in Fig. 7c. Compared with Cont rats, HFD group rats resulted in significant increase in insulin in serum (*P* < 0.01). Compared with HFD rats, levels of insulin in serum of HFD-DR-Ex and HFD-DR-Ex-MLD resulted in significant decrease (*P* < 0.01).

### The level of TNF-α, leptin and PKB in serum and liver

**Fig. 8:**
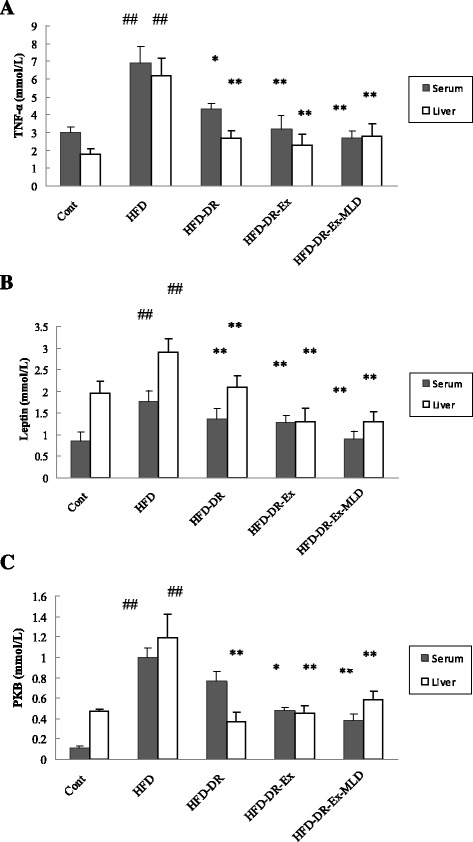
Changes of TNF-α (**a**), leptin (**b**) and PKB (**c**) in serum and liver (*n* = 10). Compared with Control group rats, ^##^ *P* < 0.01; compared with HFD rats, ^*^ *P* < 0.05, ^**^ *P* < 0.01, respectively

The level of TNF-α in serum and liver is demonstrated in Fig. 8a. Compared with Cont rats, HFD rats resulted in significant increase in TNF-α in serum and liver (*P* < 0.01). Compared with HFD rats, levels of TNF-α in serum and liver of HFD-DR, HFD-DR-Ex and HFD-DR-Ex-MLD resulted in significant decrease (*P* < 0.05 or 0.01). The level of leptin in serum and liver is demonstrated in Fig. 8b. Compared with Cont rats, HFD rats resulted in significant increase in leptin in serum and liver (*P* < 0.01). Compared with HFD rats, levels of leptin in serum and liver of HFD-DR, HFD-DR-Ex and HFD-DR-Ex-MLD resulted in significant decrease (*P* < 0.01). The level of PKB in serum and liver is demonstrated in Fig. 8c. Compared with Cont rats, HFD rats resulted in significant increase in PKB in serum and liver (*P* < 0.01). Compared with HFD rats, levels of PKB in serum and liver for HFD-DR, HFD-DR-Ex and HFD-DR-Ex-MLD rats significant decreased (*P* < 0.05 or 0.01) except serum PKB level of HFD-DR rats.

## Discussion

In past decades, studies have showed that the HFD promotes development of obesity and that there is a direct connection between dietary fat and the process of obesity. HFD has turned out to be the main factor of obesity [22]. The relationship between obesity and MS are also well demonstrated in the documents, rapidly increased body weight is also considered to be connected with MS [23–25]. There also have evidences mentioned that HFD can cause abnormal levels of leptin, insulin and PKB. In our experiment, we fed HFD to induce obesity model rats. The obesity models we made are in agreement with a series of clinical syndrome of MS, including hypertension, hyperglycemia, hyperlipaemia, hyperinsulinism and increased ratio of liver and tissue to body weight.

Our results showed that dietary restriction incorporated with aerobic exercise and MLD exhibited effects in alleviating HFD-induced obesity and dyslipidemia. Obesity plays a central role in MS, as shown in Fig. 2, HFD caused a significant increase in body weight and adipose tissue and liver mass in HFD group. These were significantly diminished by treatment, demonstrating an anti-obesity effect of dietary restriction incorporated with aerobic exercise and MLD. Moreover, compared with that of HFD rats, the serum lipid profile was normalized by treatment as serum TC, TG, LDL and FFA levels were significantly decreased and the HDL levels were significantly elevated. These findings are in agreement with our previous work showing hypolipidemic effects of dietary restriction incorporated with MLD treatment in patients [18, 19], which is largely due to increased fecal lipid excretion.

Consumption of diets rich in fats can lead to hepatic fat accumulation and liver dysfunctions [26], conditions that are underlined by leakage of cellular enzymes, such as AST, ALT and ALP [27]. In the present study, the activity of these enzymes in serum from the HFD group of rats were higher than those in the control group, which indicates that the HFD group rats had severe liver cell damage. After intervention, the activities of AST, ALT and ALP in HFD-DR-Ex-MLD group decreased significantly, demonstrating an attenuation effect on hepatic necrosis. Combining the macroscopic observation and histopathological observation of liver, we found that compared to the model control group, dietary restriction, dietary restriction incorporated with swimming exercise and dietary restriction incorporated with swimming exercise and MLD can reduce fatty deposition and decrease the degeneration in liver cells, liver of HFD-DR-Ex-MLD rats showed almost a normal liver architecture.

The endothelial dysfunction and increased BP play an important role in the development of secondary cardiovascular complications in MS [28]. Dietary restriction incorporated with exercise and MLD decreased significantly hypertension. The results indicate that intervention is very effective to prevent systolic BP increase. To our knowledge, this is the first detailed description of the hypotensive effect of MLD in HFD rats. Treatment with dietary restriction incorporated with exercise and MLD also improved parameters of type 2 diabetes. As such, glucose levels were significantly lowered by treatment. Moreover, the treatment can improve level of insulin in serum. Insulin has significant relationship with distribution of body fat. With the increase of adipose in cell population, the sensitivity of the population degrades gradually decreased, which is an abnormal cell axis feedback mechanism to produce hyperinsulinemia, however, hyperinsulinemia can further aggravate the blood lipid metabolic disorder. In our experiment, insulin serum content of the HFD group rats greatly increased. After intervention, serum insulin content in HFD-DR-Ex and HFD-DR-Ex-MLD groups decreased significantly.

To further investigate possible mechanisms of effect, some relevant cytokine, hormone and enzyme such as TNF-α, leptin and PKB were assayed simultaneously. TNF-α is always associated with obesity and other markers of inflammation [29]. Moreover, evidence turned out those levels of TNF-α in liver and serum from patients with MS who has the typical syndrome found a similar association between TNF-α and insulin resistance [30–32]. Our study demonstrates that after intervention, the serum and liver TNF-α content are significantly less in HFD-DR group, HFD-DR-Ex group and HFD-DR-Ex-MLD group rats than HFD group rats. Leptin is a hormone produced by adipocytes, leptin always control our food intake, energy metabolism, and associated with sugar and fat metabolism, decrease the level of TG, and promoted the sensitivity of liver and tissue to insulin. Leptin can improve fatty liver independently of insulin sensitization and suppression of food intake [33]. In our study, serum and liver leptin levels of the HFD group rats increased significantly. After intervention, serum and liver leptin levels in HFD-DR, HFD-DR-Ex and HFD-DR-Ex-MLD group rats decreased significantly. PKB, also known as Akt, is a serine/threonine-specific protein kinase that plays a key role in multiple cellular processes such as glucose metabolism, apoptosis, cell proliferation, transcription and cell migration; MS fails to activate PKB in cells [34, 35]. Impaired activation of PKB not only inhibits the metabolic functions of insulin, including its ability to induce glucose transport and glycogen synthesis, but it also impairs the growth-promoting and antiapoptotic functions of insulin and its related ligand the insulin-like growth factor-1 [36]. Our study demonstrates that dietary restriction incorporated with exercise or MLD significantly decreases PKB in serum and liver. These results indicated that the underlying mechanisms of dietary restriction incorporated with exercise and MLD are relevant with secretion or activation of TNF-α, leptin and PKB in serum and liver.

## Conclusions

In summary, MLD incorporated with dietary restriction and exercise treatment exhibit effects in alleviating high-fat diet-induced obesity, hyperglycemia, hyperlipidemia, hypertension, hepatic injury and insulin resistance, which are possibly due to the down-regulation of TNF-α, leptin and PKB; and this treatment can be used as a new therapeutic schedule of high-fat diet-induced MS.

## Abbreviations

ALP: Alkaline phosphatase
ALT: Glutamic-pyruvic transaminase
AST: Aspartate aminotransferase
BP: Blood pressure
ELISA: Enzyme-linked immunosorbent assay
FFA: Free fatty acid
HDL: High-density lipoprotein
HPLC: High performance liquid chromatography
LDL: Low-density lipoprotein
LZD: Linggui zhugan decoction
MLD: Modified lingguizhugan decoction
MS: Metabolic syndrome
PKB: Protein kinase B
TC: Total cholesterol
TG: Triglyceride
TNF-α: Tumor necrosis factor-α

## References

[1] Alberti KG, Zimmet P, Shaw J, IDF Epidemiology Task Force Consensus Group (2005). The metabolic syndrome-a new worldwide definition. Lancet.

[2] Ortiz MS, Myers HF, Dunkel Schetter C, Rodriguez CJ, Seeman TE (2015). Psychosocial predictors of metabolic syndrome among Latino Groups in the Multi-Ethnic Study of Atherosclerosis (MESA). PLoS One.

[3] Simons LA, Simons J, Friedlander Y, McCallum J (2011). Is prediction of cardiovascular disease and all-cause mortality genuinely driven by the metabolic syndrome, and independently from its component variables? The Dubbo study. Heart Lung Circ.

[4] Ford ES, Giles WH, Dietz WH (2002). Prevalence of the metabolic syndrome among US adults: findings from the third National Health and Nutrition Examination Survey. JAMA.

[5] Gu D, Reynolds K, Wu X, Chen J, Duan X, Reynolds RF (2005). Prevalence of the metabolic syndrome and overweight among adults in China. Lancet.

[6] Asrih M, Jornayvaz FR (2015). Metabolic syndrome and nonalcoholic fatty liver disease: is insulin resistance the link?. Mol Cell Endocrinol.

[7] Webber L, Divajeva D, Marsh T, McPherson K, Brown M, Galea G (2014). The future burden of obesity-related diseases in the 53 WHO European-Region countries and the impact of effective interventions: a modelling study. BMJ Open.

[8] Chugh PK, Sharma S (2012). Recent advances in the pathophysiology and pharmacological treatment of obesity. J Clin Pharm Ther.

[9] Caponi PW, Lehnen AM, Pinto GH, Borges J, Markoski M, Machado UF (2013). Aerobic exercise training induces metabolic benefits in rats with metabolic syndrome independent of dietary changes. Clinics (Sao Paulo).

[10] Matsuo T, So R, Shimojo N, Tanaka K (2015). Effect of aerobic exercise training followed by a low-calorie diet on metabolic syndrome risk factors in men. Nutr Metab Cardiovasc Dis.

[11] Pósa A, Szabó R, Kupai K, Csonka A, Szalai Z, Veszelka M (2015). Exercise training and calorie restriction influence the metabolic parameters in ovariectomized female rats. Oxid Med Cell Longev.

[12] Zhang JJ, Huang YJ, Ke B, Liu LP, Shangguan JJ, Meng J, et al. Effect of alternate-day fasting therapy combined with Linggui Zhugan Decoction on hepatic oxidative stress in hyperlipidemic rat. Chin J Integr Med. 2015 [Epub ahead of print]. 10.1007/s11655-014-1999-8 25588941

[13] Wang YY, Jin MH, Ke B, Li SH, Shen YZ, Zhai JY (2013). Effects of linggui zhugan decoction combined calorie restriction on the insulin resistance of model rats and mechanisms research. Chin J Integr Med.

[14] Du YM, Li XH, Huang JR, Yan J (2013). Effects of reformatted lingguizhugan decoction on body weight, blood pressure and plasma cholesterol, triacylglycerol, high density lipoprotein and low density lipoprotein in metabolic syndrome model rats. Zhongyao Yaoli Yu Linchuang.

[15] Huang JR, Li XH, Du YM, Yan J, Hu Y (2013). Effects of reformatted Lingguizhugan Decoction on the glycometabolism disorder in metabolic syndrome model rats. Zhongyao Yaoli Yu Linchuang.

[16] Huang JR, Du YM, Yan J, Hu Y, Li XH (2013). Effects of reformatted Linggui Zhugan Decoction on the level of Serum Resistin, Adiponectin, insulin and insulin resistance in metabolic syndrome model rats. Chin J Exp Tradit Med Formulae.

[17] Michalsen A, Hoffmann B, Moebus S, Bäcker M, Langhorst J, Dobos GJ (2005). Incorporation of fasting therapy in an integrative medicine ward: evaluation of outcome, safety, and effects on life-style adherence in a large prospective cohort study. J Altern Complement Med.

[18] Chen DS, Ke B, Huang YJ, Meng J, Zhang JJ, Chen ZX (2011). Effects of the modified linggui zhugan decoction (see text) combined with short-term very low calorie diets on glycemic control in newly diagnosed type 2 diabetics. J Tradit Chin Med.

[19] Ke B, Shi L, Jun-jie Z, Chen DS, Meng J, Qin J (2012). Protective effects of modified linggui zhugan decoction combined with short-term very low calorie diets on cardiovascular risk factors in obese patients with impaired glucose tolerance. J Tradit Chin Med.

[20] Sinitskaya N, Gourmelen S, Schuster-Klein C, Guardiola-Lemaitre B, Pévet P, Challet E (2007). Increasing the fat-to-carbohydrate ratio in a high-fat diet prevents the development of obesity but not a prediabetic state in rats. Clin Sci (Lond).

[21] Chen H, Chiang IP, Jen CJ (1996). Exercise training increases acetylcholine-stimulated endothelium-derived nitric oxide release in spontaneously hypertensive rats. J Biomed Sci.

[22] Hariri N, Thibault L (2010). High-fat diet-induced obesity in animal models. Nutr Res Rev.

[23] Hirahatake KM, Slavin JL, Maki KC, Adams SH (2014). Associations between dairy foods, diabetes, and metabolic health: potential mechanisms and future directions. Metabolism.

[24] Thamer C, Machann J, Stefan N, Haap M, Schäfer S, Brenner S (2007). High visceral fat mass and high liver fat are associated with resistance to lifestyle intervention. Obesity (Silver Spring).

[25] Lin CY, Chen PC, Kuo HK, Lin LY, Lin JW, Hwang JJ (2010). Effects of obesity, physical activity, and cardiorespiratory fitness on blood pressure, inflammation, and insulin resistance in the National Health and Nutrition Survey 1999–2002. Nutr Metab Cardiovasc Dis.

[26] Panchal SK, Poudyal H, Iyer A, Nazer R, Alam A, Diwan V (2011). High-carbohydrate high-fat diet–induced metabolic syndrome and cardiovascular remodeling in rats. J Cardiovasc Pharmacol.

[27] Poudyal H, Campbell F, Brown L (2010). Olive leaf extract attenuates cardiac, hepatic, and metabolic changes in high carbohydrate-, high fat-fed rats. J Nutr.

[28] Jabir NR, Firoz CK, Khan MS, Zaidi SK, Ashraf GM, Shakil S, et al. Potential linkage between cerebrovascular diseases and metabolic syndrome. Curr Drug Metab. 2016 [Epub ahead of print]. 10.2174/1389200217666160810155055 27515564

[29] Möller K, Ostermann AI, Rund K, Thoms S, Blume C, Stahl F (2016). Influence of weight reduction on blood levels of C-reactive protein, tumor necrosis factor-α, interleukin-6, and oxylipins in obese subjects. Prostaglandins Leukot Essent Fatty Acids.

[30] Lechleitner M, Herold M, Dzien-Bischinger C, Hoppichler F, Dzien A (2002). Tumour necrosis factor-alpha plasma levels in elderly patients with Type 2 diabetes mellitus-observations over 2 years. Diabet Med.

[31] Carey AL, Bruce CR, Sacchetti M, Anderson MJ, Olsen DB, Saltin B (2004). Interleukin-6 and tumor necrosis factor-alpha are not increased in patients with Type 2 diabetes: evidence that plasma interleukin-6 is related to fat mass and not insulin responsiveness. Diabetologia.

[32] Khosravi R, Ka K, Huang T, Khalili S, Nguyen BH, Nicolau B (2013). Tumor necrosis factor-α and interleukin-6: potential interorgan inflammatory mediators contributing to destructive periodontal disease in obesity or metabolic syndrome. Mediators Inflamm.

[33] Yamamoto-Kataoka S, Ebihara K, Aizawa-Abe M, Nishio M, Kusakabe T, Yamamoto Y (2015). Leptin improves fatty liver independently of insulin sensitization and appetite suppression in hepatocyte-specific Pten-deficient mice with insulin hypersensitivity. Horm Metab Res.

[34] Manning BD (2004). Balancing Akt with S6K: implications for both metabolic diseases and tumorigenesis. J Cell Biol.

[35] Farese RV, Sajan MP, Standaert ML (2005). Insulin-sensitive protein kinases (atypical protein kinase C and protein kinase B/Akt): actions and defects in obesity and type II diabetes. Exp Biol Med (Maywood).

[36] Guvakova MA (2007). Insulin-like growth factors control cell migration in health and disease. Int J Biochem Cell Biol.

